# Comparative Analysis of Surface Modification Techniques for Assessing Oral Implant Osseointegration: An Animal Study

**DOI:** 10.7759/cureus.54014

**Published:** 2024-02-11

**Authors:** Karthik K S, B Sreevidya, Ramya T K, Divya BM, N R Dedeepya, Bhumika Kamal Badiyani, Amit Kumar

**Affiliations:** 1 Department of Prosthodontics and Implantology, KGF (Kolar Gold Fields) College of Dental Sciences, Kolar, IND; 2 Department of Oral and Maxillofacial Surgery, KGF (Kolar Gold Fields) College of Dental Sciences, Kolar, IND; 3 Department of Oral Medicine and Radiology, KGF (Kolar Gold Fields) College of Dental Sciences, Kolar, IND; 4 Department of Public Health Dentistry, D. A. Pandu Memorial RV (Rashtreeya Vidyalaya) Dental College, Bangalore, IND; 5 Department of Public Health Dentistry, Interdental Multispeciality Dental Clinic, Mumbai, IND

**Keywords:** implantology, dentistry, surface modifications, osseointegration, implants

## Abstract

Background: Effective implant placement depends critically on the implant's level of osseointegration with the alveolar bone. To increase osseointegration during implant placement, research has concentrated on the surface modification of implants, and morphological analyses have looked at the thread pattern in close interaction with the bone's surface.

Aim: This study aimed to assess and compare the extent of oral implant osseointegration in different surface modification techniques.

Materials and methods: In this study, 12 healthy adult dogs aged 18-24 months were used. Tooth extractions were performed on both sides of the mandible, and wounds were closed with sutures. Two months later, the right mandible of each dog underwent local anesthesia and general anesthesia. Four different implant types were placed based on their surface treatments: resorbable blast media (RBM)-treated implants, hydroxyapatite (HA) implants with an ultra-thin HA film, hydrothermal-treated HA implants coated with HA, and sandblasting combined acid etching (SLA) implants treated with plasma spray and acid etching. A total of 48 implants were divided into two- and four-week groups, with identical dimensions. Each dog received two implants from each group, for a total of eight implants per dog. The implants were securely placed into the superior alveolar bone with a torque greater than 35-N up to a depth of 1 mm. Periotest M (Medizintechnik Gulden e.K., Modautal, Germany) was used to calculate the periotest value (PTV) as a typical value on the buccal side of each implant immediately following placement and sacrifice to test the main fixation and stability of the implants. Resonance frequency analysis (RFA) was utilised by Osstell Mentor (Osstell AB, Gothenburg, Sweden) to simultaneously assess the implant stability quotient (ISQ) on the medial, distal, buccal, and lingual sides of the implant. The rotational torque in one of the sacrificed dogs was calculated using the MGT 50 (ELECTROMATIC Equipment Co., Inc., New York, USA) torque analyzer. The histomorphometric evaluation was performed using an optical microscope (Olympus Corporation, Tokyo, Japan). The upper half's bone-implant contact (BIC), which was found to be more important for implant stability, was studied together with the ratio of the new bone formation area (NBFA) to the complete implant.

Results: The maximum stability was observed in HA-treated implants in the fourth week. The minimum stability was observed in hydrothermal-treated HA implants in the fourth week. The stability in each group was greater in the four-week evaluation as compared to the two-week evaluation. The stability was satisfactory in almost all implants at two- and three-week evaluations. The maximum value of the percentage area of newly formed bone at the two- and four-week evaluations was observed in HA-treated implants. The minimum value of the percentage of the area of newly formed bone at two- and four-week evaluations was observed in SLA and RBM-treated implants respectively. The difference was significant statistically (p ≤ 0.05).

Conclusion: All implant surface modifications, in general, produced satisfactory osseointegration. Excellent osseointegration was seen in the upper portion of the implant with hydrothermally treated HA.

## Introduction

A frequent therapeutic strategy is to use an implant to restore an edentulous region. To accomplish preliminary and permanent implant placement success, a number of prerequisites must be met. One of the requirements is for the implant along with the alveolar bone to have strong osseointegration [1-3]. The material, design, and interface of the dental implant, as well as the quality of the bone at the placement site, the surgical technique, and the loading circumstances are just six of the crucial factors that Albrektsson et al. proposed influence osseointegration of implants [4,5]. Research has been carried out to improve implant surfaces for the last two decades, and various issues including chemical processing and physical characteristics, such as the surface's roughness, have been examined [6].

Effective implant placement depends critically on the implant's level of osseointegration with the alveolar bone [7-12]. To increase osseointegration during implant placement, research has concentrated on the surface modification of implants, and morphological analyses have looked at the thread pattern in close interaction with the bone's surface [13]. The morphological alongside chemical characteristics of the implant, such as the charge of the surface, protein absorption, wettability, cell and tissue growth on the surface, and cell-surface interactions according to Ratner and Porter [14], are significant factors that influence how the surface interacts with the bioactive materials. According to Davies' research [15], platelet activation at the outermost layer of the implant leads to an interaction between osteocytes and the implant interface that promotes the production of new bone and increases bone conduction continuity.

To achieve a smooth surface, the conventional Brånemark titanium implant is physically polished. However, many writers have looked into the uses of coarse interfaces as it was suggested that dental implants with a rough surface would be more effective in spongy bones with low bone quality [16,17]. A technique called titanium plasma spray (TPS) is utilized to create a rough surface and enhance the actual bone interaction space.

Hydroxyapatite (HA), calcium phosphate (CaP), titanium dioxide (TiO_2_), and aluminum oxide (Al_2_O_3_) with good biocompatibility, are used in a plasma spray surface modification that is currently attracting increasing attention [9]. Furthermore, the outermost layer can become rougher and encourage the development of osteoblasts through acid etching using nitric acid (HNO_3_), hydrogen chloride (HCl), and sulfuric acid (H_2_SO_4_). Sandblasting combined acid etching (SLA) has been used in a few investigations [9,10]. Anodic oxidation or fluorination to boost osseointegration is also being researched for implant placement [11].

When the implant was given four distinct surface modifications-resorbable blast media (RBM), which has currently come into commercial use; HA coating; acid etching accompanied by plasma spray; and hydrothermally treated HA, to increase the extent of particle adherence-we carried out histomorphometric assessments of implant osseointegration.

## Materials and methods

Twelve healthy adult dogs between the ages of 18 and 24 months were used in the tests. Each dog received an intramuscular injection in the thigh containing a muscle relaxant (2% xylazine hydrochloride, 10 mg/kg, Rompun Inj.; Bayer AG, Leverkusen, Germany), an anaesthetic (tiletamine, zolazepam, 10 mg/kg, Zoletil 50; Virbac Lab, Carros, France), and 2% hydrochloride lidocaine (Yuhan Co., Ltd.). On both sides, the first and second mandibular premolars and molars were extracted. Vicryl in size 4-0 (Ethicon, Inc., Raritan, New Jersey, United States) was used to close wounds. The study was approved by the Institutional Ethics Committee of KGF (Kolar Gold Fields) College of Dental Sciences (approval number: IEC/KGF/2021/11).

Two months after tooth extraction, each animal's right mandible received local anesthesia treatment before being put under general anaesthesia. The four different implant types were placed according to how their surfaces had been treated: RBM-treated implants (Clearant; Dentis Inc., Daegu, Korea), HA implants covered in an ultra-thin HA film at room temperature (Haptite; Dentis Inc.), hydrothermal-treated HA implants coated with HA (Dentis Inc.), and SLA implants treated with plasma spray and acid etching (One Q SL; Dentis Inc.). The 48 implants were divided into two- and four-week groups and had identical lengths and diameters (3.7-8.0 mm). Each dog received two implants (a total of eight implants) from each group. Additionally, each implant was implanted with a torque greater than 35-N up to a depth of 1 mm on the superior alveolar bone.

Suxamethonium chloride hydrate (0.11 mg/kg, Eagle Suxamethonium Inj., Korea) was used to kill the animals. The mandibles were removed immediately after sacrifice, formalin-preserved for 10 days, and then embedded in glycol-methacrylate resin (Spurr low-viscosity embedding media, USA). The specimens were polished to a thickness of 50 m or less using a lapping and polishing machine (OMNILAP 2000, USA) and were sliced using a high-precision diamond disc. Villanueva Osteochrome Bone Stain (SBT), which was used to prepare the histology specimens, was the last step.

Periotest M was used to calculate the periotest value (PTV) as a typical value on the buccal side of each implant immediately following placement and sacrifice to test the main fixation and stability of the implants. Resonance frequency analysis (RFA) was utilized by the Osstell Mentor (Sweden) to simultaneously assess the implant stability quotient (ISQ) on the medial, distal, buccal, and lingual sides of the implant. The rotational torque in one of the sacrificed dogs was calculated using the MGT 50 (ELECTROMATIC Equipment Co., Inc., New York, USA) torque analyzer. The histomorphometric evaluation was performed using an optical microscope (Olympus, Japan). The upper half’s bone-implant contact (BIC), which was found to be more important for implant stability, was studied together with the ratio of the new bone formation area (NBFA) to the complete implant.

Statistical analysis: Each estimate was examined using PASW Statistics, Version 18.0 (IBM Corp., Armonk, New York, United States). One-way ANOVA was used to evaluate statistical significance, and the statistical evaluation was done with a 95% confidence level.

## Results

The values of ISQ in RBM implants at baseline were 69.4±7.5. The values of ISQ in RBM at the two-week evaluation were 76.7±8.6. The values of ISQ in RBM at the four-week evaluation were 79.9±4.92. The values of ISQ in HA-treated implants at baseline were 67.3±10.0. The values of ISQ in HA-treated implants at the two-week evaluation were 74.4 ±7.2. The values of ISQ in HA-treated implants at the four-week evaluation were 80.7±4.93. The values of ISQ in hydrothermally treated HA implants at baseline were 69.0±11.7. The values of ISQ in hydrothermally treated HA implants at two weeks of evaluation were 73.2±6.71. The values of ISQ in hydrothermally treated HA implants in the fourth week of evaluation were 75.9 ±4.72. The values of ISQ in SLA-treated implants at baseline were 74.6±9.54. The values of ISQ in SLA-treated implants at the two-week evaluation were 73.9 ±4.83. The values of ISQ in SLA-treated implants in the fourth week of evaluation were 76.8±2.92. The maximum stability was observed in HA-treated implants in the fourth week. The minimum stability was observed in hydrothermally treated HA implants in the fourth week. The stability in each group was greater at four weeks of evaluation as compared to two weeks of evaluation. The stability was satisfactory in almost all implants at the two-week and four-week evaluations. (Table 1)

**Table 1:** Implant stability quotient based on resonance frequency analysis RBM: Resorbable blast media; HA: Hydroxyapatite; SLA: Sandblasting combined acid etching

Surface treatment	RBM	HA	Hydrothermal-treated HA	SLA
Baseline	69.4±7.5	67.3±10.0	69.0±11.7	74.6±9.54
2 week	76.7±8.6	74.4 ±7.2	73.2±6.71	73.9 ±4.83
4 week	79.9 ±4.92	80.7±4.93	75.9 ±4.72	76.8±2.92

The total range of ISQ was 1 to 100.

The values of periotest in RBM implants at baseline were -5.46±0.55. The value of the periotest in RBM at the two-week evaluation was -4.64±1.98. The values of periotest in RBM in the fourth week of evaluation were -5.53±0.79. The values of periotest in HA-treated implants at baseline were -5.21 ± 0.72. The values of periotest in HA-treated implants at two weeks of evaluation were -4.44±2.17. The values of periotest in HA-treated implants in the fourth week of evaluation were -5.33±0.67. The values of periotest in hydrothermally treated HA implants at baseline were -5.64±0.61. The values of periotest in hydrothermally treated HA implants at two weeks of evaluation were -5.13±0.73. The values of periotest in hydrothermally treated HA implants in the fourth week of evaluation were -5.44±1.16. The values of periotest in SLA-treated implants at baseline were -6.18 ± 0.37. The values of periotest in SLA-treated implants at two weeks of evaluation were -5.23±1.11. The values of periotest in SLA-treated implants in the fourth week of evaluation were -6.18±0.43. (Table 2)

**Table 2:** Comparison of PTV for different surface treatments over time RBM: Resorbable blast media; HA: Hydroxyapatite; SLA: Sandblasting combined acid etching; PTV: Periotest values

Surface treatment	RBM	HA	Hydrothermal-treated HA	SLA
Initial	–5.46±0.55	–5.21 ±0.72	–5.64±0.61	–6.18±0.37
2 week	–4.64±1.98	–4.44±2.17	–5.13±0.73	–5.23±1.11
4 week	–5.53±0.79	–5.33±0.67	–5.44±1.16	–6.18 ±0.43

The total range of periotest was -8 to +50.

The normal torque values for successful osseointegration have been reported to range between 25 N/cm to 50 N/cm. The value of torque for removal in RBM at the two-week evaluation was 83.9. The values of torque for removal in RBM at the four-week evaluation were greater than 135. The value of torque for removal of HA-treated implants at the two-week evaluation was 101.8. The values of torque for the removal of HA-treated implants in the fourth week of evaluation were greater than 135. The values of torque for removal of hydrothermally treated HA implants at two weeks of evaluation were 93.2. The values of torque for removal in hydrothermally treated HA implants in the fourth week of evaluation were greater than 135. The value of torque for removal of SLA-treated implants at the two-week evaluation was 93.0. The values of torque for the removal of SLA-treated implants in the fourth week of evaluation were greater than 135. It was observed that the maximum values of torque for the removal of implants were for HA-treated implants at two weeks, while the minimum values were for RBM-treated implants. (Table 3)

**Table 3:** Removal torque values (N/cm) for different surface treatments at two weeks and four weeks RBM: Resorbable blast media; HA: Hydroxyapatite; SLA: Sandblasting combined acid etching

Surface treatment	RBM	HA	Hydrothermal-treated HA	SLA
2 week	83.9	101.8	93.2	93.0
4 week	135	135	135	135

The percentage of the area of new bone formed in RBM at two weeks of evaluation was 58.6±12.5. The percentage of the area of new bone formed in RBM in the fourth week of evaluation was 63.6±5.4. The percentage of the area of new bone formed in HA-treated implants at two weeks of evaluation was 64.6±23.7. The percentage of the area of new bone formed in HA-treated implants in the fourth week of evaluation was 75.1±16.2. The percentage of the area of new bone formed in Hydrothermal-treated HA implants at two weeks of evaluation was 62.6±13.8. The percentage of the area of new bone formed in Hydrothermal-treated HA implants in the fourth week of evaluation was 71.4±13.7. The percentage of the area of new bone formed in SLA-treated implants at two weeks of evaluation was 52.4±29.9. The percentage of the area of new bone formed in SLA-treated implants in the fourth week of evaluation was 66.4±13.6. The maximum value of the percentage area of newly formed bone at the two- and four-week evaluations was observed in HA-treated implants. The minimum value of the percentage of area of newly formed bone at the two- and four-week evaluations was observed in SLA and RBM-treated implants, respectively. The difference was significant statistically (p ≤ 0.05). (Table 4)

**Table 4:** NBFA ratios (%) for different surface treatments at two weeks and four weeks RBM: Resorbable blast media; HA: Hydroxyapatite; SLA: Sandblasting combined acid etching; NBFA: New bone formation area

Surface treatment	RBM	HA	Hydrothermal-treated HA	SLA	P value
2 week	58.6 ±12.5	64.6 ±23.7	62.6 ±13.8	52.4 ±29.9	0.001
4 week	63.6 ±5.4	75.1 ±16.2	71.4±13.7	66.4±13.6	

At two weeks, 64.9±4.3 of new bone was in contact with the RBM implant. At four weeks, 67.4±3.9 of new bone was in contact with the RBM implant. At two weeks, 65.6±25.5 of new bone was in contact with HA-treated implants. At four weeks, 73.9±18.6 of new bone was in contact with HA-treated implants. The percentage of new bone in contact with the implant in SLA-treated implants at two weeks of evaluation was 61.9±27.1. The percentage of new bone in contact with the implant in SLA-treated implants in the fourth week of evaluation was 72.4±9.4. The maximum value of the percentage of new bone in contact with the implant at the two- and four-week evaluations was observed in HA-treated implants. The minimum value of the percentage of new bone in contact with the implant at the two- and four-week evaluations was observed in SLA and RBM-treated implants, respectively. The difference was significant statistically (p ≤ 0.05). (Table 5)

**Table 5:** BIC ratios (%) for different surface treatments at two weeks and four weeks BIC: bone-implant contact

Surface treatment	RBM	HA	Hydrothermal-treated HA	SLA	P value
2 week	64.9 ±4.3	65.6 ±25.5	67.4±13.5	61.9±27.1	0.02
4 week	67.4 ±3.9	73.9±18.6	74.1±12.1	72.4±9.4	

## Discussion

The degree of osseointegration between the implant and the alveolar bone is crucial for successful implant placement. Research has focused on the surface modification of implants to improve osseointegration during implant insertion, and morphological investigations have examined the thread pattern in close contact with the surface of the bone. The morphological and chemical properties of the implant, including the surface charge, protein absorption, wettability, cell and tissue growth on the surface, and cell-surface interactions, are important factors that influence how the surface interacts with the bioactive materials. According to previous research, platelet activation at the implant's outermost layer causes osteocytes and the implant's interface to interact in a way that encourages the growth of new bone and lengthens bone conduction continuity [15-18]. When the implant was given four distinct surface modifications-RBM, which has currently come into commercial use, HA coating, acid etching accompanied by plasma spray, and hydrothermally treated HA, to increase the extent of particle adherence-we carried out histomorphometric assessments of implant osseointegration.

The maximum value of the percentage area of newly formed bone at the two- and four-week evaluations was observed in HA-treated implants. The minimum value of the percentage of area of newly formed bone at the two- and four-week evaluations was observed in SLA and RBM-treated implants, respectively. The difference was significant statistically (p ≤ 0.05). The percentage of new bone formed in RBM at the two-week evaluation was 58.6±12.5. The percentage of new bone formed in RBM in the fourth week of evaluation was 63.6±5.4. The percentage of new bone formed in HA-treated implants at the two-week evaluation was 64.6± 23.7. The percentage of new bone formed in HA-treated implants at the four-week evaluation was 75.1±16.2.

The percentage of new bone formed in hydrothermally treated HA implants at two weeks of evaluation was 62.6±13.8. The percentage of new bone formed in hydrothermally treated HA implants in the fourth week of evaluation was 71.4±13.7. The percentage of new bone formed in SLA-treated implants at the two-week evaluation was 52.4±29.9. The percentage of new bone formed in SLA-treated implants at the four-week evaluation was 66.4±13.6. The restoration of an edentulous area using an implant is a common treatment technique. Several conditions must be satisfied for the preliminary and permanent implant placement to be successful. Strong osseointegration between the implant and alveolar bone is one need. The quality of the bone at the implantation site, the surgical procedure, the loading conditions, and the material, design, and interface of the dental implant are just six of the critical aspects Albrektsson et al. claimed impact osseointegration of implants [4]. For the past 20 years, studies have been conducted to enhance implant surfaces and a variety of topics including chemical processing, and physical properties such as surface roughness have been investigated [19-21].

The typical Brånemark titanium implant is physically polished to produce a flat surface. Though it was hypothesized that dental implants with a rough surface would be more effective in spongy bones with low bone quality, many writers have looked at the use of coarse interfaces [22,23]. To make a rough surface and improve the actual bone interaction space, TPS is used. A plasma spray surface modification that is currently receiving more attention uses HA, CaP, TiO_2_, and Al_2_O_3_ with good biocompatibility [9]. Furthermore, using HNO_3_, HCl, and H_2_SO_4_ to etch the outermost layer might make it rougher and promote the growth of osteoblasts. In a few studies [9,10], SLA has been utilized. Also being investigated for implant implantation are anodic oxidation or fluorination to promote osseointegration [24,25].

In this study, the percentage of new bone in contact with the implant in RBM at the two-week evaluation was 64.9±4.3. The percentage of new bone in contact with the implant in RBM at the four-week evaluation was 67.4±3.9. The percentage of new bone in contact implants in HA-treated implants at two weeks of evaluation was 65.6 ±25.5. The percentage of new bone in contact of implants in HA-treated implants in the fourth week of evaluation was 73.9±18.6. The percentage of new bone in contact with the implant in hydrothermally treated HA implants at the two-week evaluation was 67.4±13.5. The percentage of new bone in contact with the implant in hydrothermally treated HA implants in the fourth week of evaluation was 74.1±12.1. The percentage of new bone in contact with the implant in SLA-treated implants at the two-week evaluation was 61.9±27.1. The percentage of new bone in contact with the implant in SLA-treated implants at the four-week evaluation was 72.4±9.4. In this study, the maximum value of the percentage of new bone in contact with the implant at the two- and four-week evaluations was observed in HA-treated implants. The minimum value of the percentage of new bone in contact with the implant at the two-week and four-week evaluations was observed in SLA and RBM-treated implants, respectively. The difference was significant statistically (p ≤ 0.05).

The current study focuses on the comparative analysis of surface modification techniques for assessing oral implant osseointegration in dogs. Human and canine bone mineralization does have some notable differences. Firstly, human bone density is generally higher than that of dogs, with denser and differently structured bone tissue, making it more resistant to mechanical stress. Additionally, the anatomy of human and canine jaws differs significantly, affecting surgical implant placement and bone interaction. Furthermore, humans and dogs exhibit distinct chewing patterns and bite forces, leading to variations in biomechanical forces acting on dental implants and impacting their stability and osseointegration. The rate of bone turnover, bone healing response, and established clinical protocols further contribute to differences between the two species. Consequently, while valuable insights can be gained from animal studies like those involving dogs, translation to human clinical practice requires careful consideration of these anatomical, physiological, and biomechanical distinctions. Further research, including clinical trials in humans, is essential to confirm the applicability of surface modification techniques in human dental implantology while accounting for the unique characteristics of human bone tissue.

This study has several limitations that need to be considered when interpreting its findings. First, the use of healthy adult dogs as an animal model, while valuable for initial insights, may not fully replicate the complexities of human bone healing and response to implants. Differences in anatomy and physiology between dogs and humans could impact the generalizability of the results to clinical practice. Dogs and humans differ significantly in size, dental structure, and overall skeletal anatomy, particularly in the jaw region. The canine jaw's size and shape diverge from human counterparts, impacting implant placement and load distribution. Furthermore, the biomechanics of jaw movement, chewing patterns, and bite forces vary between species, affecting the stress and strain experienced by dental implants during function. Typically, humans exhibit higher bone density, especially in load-bearing areas like the jawbone. These differences can influence implant stability and the osseointegration process, which relies on the quality and quantity of available bone. Second, the short-term nature of the evaluation, with assessments at two weeks and four weeks, provides valuable information about early osseointegration but lacks insights into the long-term stability and durability of implant integration, which is crucial for assessing clinical success. Third, the study's relatively small sample size, involving only 12 dogs, may limit the statistical power and generalizability of the findings. Expanding the sample size could enhance the reliability of the results. Additionally, the surgical expertise and technique used for implant placement could vary among surgeons, potentially introducing bias into the outcomes. Further clinical research is essential to confirm the applicability of these surface modification techniques in human dental implantology.

## Conclusions

All implant surface modifications, in general, produced satisfactory osseointegration. Excellent osseointegration was seen in the upper portion of the implant with hydrothermally treated HA. Further study is necessary to combine the current implant surface treatment techniques with modified and upgraded technologies to enhance osseointegration.

The study not only highlights the overall positive outcomes of different surface modification techniques for osseointegration but also specifically identifies the promise of hydrothermally treated HA implants. It emphasizes the importance of continued research and development in implant dentistry to refine and advance surface modification approaches, ultimately benefiting patients seeking reliable and stable dental implant solutions.

## References

[1] Mueller CK, Thorwarth M, Schmidt M, Schlegel KA, Schultze-Mosgau S (2011). Comparative analysis of osseointegration of titanium implants with acid-etched surfaces and different biomolecular coatings. Oral Surg Oral Med Oral Pathol Oral Radiol Endod.

[2] Abuhussein H, Pagni G, Rebaudi A, Wang HL (2010). The effect of thread pattern upon implant osseointegration. Clin Oral Implants Res.

[3] de Vicente JC, Recio O, Martín-Villa L, Junquera LM, López-Arranz JS (2006). Histomorphometric evaluation of guided bone regeneration around implants with SLA surface: an experimental study in beagle dogs. Int J Oral Maxillofac Surg.

[4] Albrektsson T, Brånemark PI, Hansson HA, Lindström J (1981). Osseointegrated titanium implants. Requirements for ensuring a long-lasting, direct bone-to-implant anchorage in man. Acta Orthop Scand.

[5] Brånemark PI, Adell R, Breine U, Hansson BO, Lindström J, Ohlsson A (1969). Intra-osseous anchorage of dental prostheses. I. Experimental studies. Scand J Plast Reconstr Surg.

[6] Shalabi MM, Gortemaker A, Van't Hof MA, Jansen JA, Creugers NH (2006). Implant surface roughness and bone healing: a systematic review. J Dent Res.

[7] Thompson JI, Gregson PJ, Revell PA (1999). Analysis of push-out test data based on interfacial fracture energy. J Mater Sci Mater Med.

[8] Buser D, Schenk RK, Steinemann S, Fiorellini JP, Fox CH, Stich H (1991). Influence of surface characteristics on bone integration of titanium implants. A histomorphometric study in miniature pigs. J Biomed Mater Res.

[9] Le Guéhennec L, Soueidan A, Layrolle P, Amouriq Y (2007). Surface treatments of titanium dental implants for rapid osseointegration. Dent Mater.

[10] Buser D, Broggini N, Wieland M (2004). Enhanced bone apposition to a chemically modified SLA titanium surface. J Dent Res.

[11] Ellingsen JE, Johansson CB, Wennerberg A, Holmén A (2004). Improved retention and bone-tolmplant contact with fluoride-modified titanium implants. Int J Oral Maxillofac Implants.

[12] Jeong R, Marin C, Granato R, Suzuki M, Gil JN, Granjeiro JM, Coelho PG (2010). Early bone healing around implant surfaces treated with variations in the resorbable blasting media method. A study in rabbits. Med Oral Patol Oral Cir Bucal.

[13] Meredith N, Book K, Friberg B, Jemt T, Sennerby L (1997). Resonance frequency measurements of implant stability in vivo. A cross-sectional and longitudinal study of resonance frequency measurements on implants in the edentulous and partially dentate maxilla. Clin Oral Implants Res.

[14] Yoon WJ, Kim SG, Oh JS, You JS, Jeong KI, Lim SC, Jeong MA (2016). Comparative study on the osseointegration of implants in dog mandibles according to the implant surface treatment. J Korean Assoc Oral Maxillofac Surg.

[15] Davies JE (2003). Understanding peri-implant endosseous healing. J Dent Educ.

[16] Schwarz ML, Kowarsch M, Rose S, Becker K, Lenz T, Jani L (2009). Effect of surface roughness, porosity, and a resorbable calcium phosphate coating on osseointegration of titanium in a minipig model. J Biomed Mater Res A.

[17] Olivé J, Aparicio C (1990). Periotest method as a measure of osseointegrated oral implant stability. Int J Oral Maxillofac Implants.

[18] Coelho PG, Granjeiro JM, Romanos GE (2009). Basic research methods and current trends of dental implant surfaces. J Biomed Mater Res B Appl Biomater.

[19] Piattelli M, Scarano A, Paolantonio M, Iezzi G, Petrone G, Piattelli A (2002). Bone response to machined and resorbable blast material titanium implants: an experimental study in rabbits. J Oral Implantol.

[20] Granato R, Marin C, Suzuki M, Gil JN, Janal MN, Coelho PG (2009). Biomechanical and histomorphometric evaluation of a thin ion beam bioceramic deposition on plateau root form implants: an experimental study in dogs. J Biomed Mater Res B Appl Biomater.

[21] Yang GL, He FM, Yang XF, Wang XX, Zhao SF (2008). Bone responses to titanium implants surface-roughened by sandblasted and double etched treatments in a rabbit model. Oral Surg Oral Med Oral Pathol Oral Radiol Endod.

[22] Cochran DL, Schenk RK, Lussi A, Higginbottom FL, Buser D (1998). Bone response to unloaded and loaded titanium implants with a sandblasted and acid-etched surface: a histometric study in the canine mandible. J Biomed Mater Res.

[23] Block MS, Kent JN, Kay JF (1987). Evaluation of hydroxylapatite-coated titanium dental implants in dogs. J Oral Maxillofac Surg.

[24] Jones JD, Lupori J, Van Sickels JE, Gardner W (1999). A 5-year comparison of hydroxyapatite-coated titanium plasma-sprayed and titanium plasma-sprayed cylinder dental implants. Oral Surg Oral Med Oral Pathol Oral Radiol Endod.

[25] Cook SD, Baffes GC, Palafox AJ, Wolfe MW, Burgess A (1992). Torsional stability of HA-coated and grit-blasted titanium dental implants. J Oral Implantol.

